# Usefulness of serum amyloid A for the diagnosis of pyelonephritis in cats: A prospective evaluation

**DOI:** 10.1111/jvim.17082

**Published:** 2024-04-26

**Authors:** Maxime Kurtz, Pascaline Bénédicte Marie Pey, Jérémy Mortier, Mathieu Manassero, Fiona Da Riz, Morgane Canonne‐Guibert, Christelle Maurey, Ghita Benchekroun

**Affiliations:** ^1^ École Nationale Vétérinaire d'Alfort, CHUVA Service de Médecine Interne Maisons‐Alfort France; ^2^ Antech Imaging Services Irvine California USA; ^3^ Veterinary Hospital “I Portoni Rossi,” Anicura, Zola Predosa (BO) Bologna Italy; ^4^ Ecole Nationale Vétérinaire d'Alfort, CHUVA Service D'Imagerie Médicale Maisons‐Alfort France; ^5^ École Nationale Vétérinaire d'Alfort, CHUVA Service de Chirurgie Maisons‐Alfort France; ^6^ Ecole Nationale Vétérinaire d'Alfort Univ Paris Est Créteil, INSERM, IMRB Maisons‐Alfort France

**Keywords:** antibiotics, biomarker, pelvic cavity, renal, urinary tract infection

## Abstract

**Background:**

The diagnosis of pyelonephritis in cats is challenging and development of a noninvasive and accurate biomarker is needed.

**Hypotheses:**

Serum amyloid A (SAA) is increased in cats with pyelonephritis, but not in cats with other urinary tract diseases.

**Animals:**

A cohort of 125 cats (149 observations).

**Methods:**

This was a prospective study. Group 1 included cats with a diagnosis of pyelonephritis either confirmed by bacterial culture of pelvic urine (Group 1a) or presumed (1b). Group 2 included cats for which pyelonephritis was ruled out (with certainty: Group 2a or judged unlikely: Group 2b). SAA concentration was compared between groups, and accuracy of SAA for the diagnosis of pyelonephritis was calculated using a Receiver Operating Characteristic (ROC) curve analysis.

**Results:**

Median SAA concentration was significantly higher in Group 1a (86.8 mg/L [73.3; 161.5]; n = 8) than in Group 2a (4 mg/L [1.8; 5.6], n = 19; *P* < .001) and in Group 2b (5.4 mg/L [3.1; 9.7], n = 113; *P* < .001). It was also significantly higher in Group 1b (98.8 mg/L [83.1; 147.3]; n = 9) than in Group 2b (*P* < .001) and Group 2a (*P* < .001). Optimal diagnostic cut‐off for SAA concentration was 51.3 mg/L. yielding a sensitivity of 88% (95% confidence interval: [64%; 99%]) and a specificity of 94% (95% confidence interval: [88%; 97%]).

**Conclusions and Clinical Importance:**

Measurement of SAA could be used to rule out pyelonephritis in the case of low suspicion of the disease. Increased SAA concentration is suggestive of pyelonephritis despite a lack of specificity.

**Abbreviations**
AKI: acute kidney injury
AUC: area under the curve
CKD: chronic kidney disease
CRP: C‐reactive protein
IF: intact female
IM: intact male
NF: neutered female
NM: neutered male
NPV: negative predictive value
PPV: positive predictive value
PUPD: polyuria and polydipsia
ROC: receiver operating characteristics
SAA: serum amyloid A
SUB: subcutaneous ureteral bypass

## 1 INTRODUCTION

The veterinary literature regarding bacterial pyelonephritis in cats is sparse, despite the disease being commonly recognized as a relevant cause of acute‐on‐chronic kidney injury.^1^ More specifically, its diagnostic features are not well defined. The diagnosis relies on the association of suggestive clinical and paraclinical abnormalities (eg, fever, azotemia, bacteriuria, painful abdominal palpation, neutrophilic leukocytosis), as well as ultrasonographic abnormalities (eg, renal pelvic dilation).^2^ However, these signs are often subjective, equivocal, and nonspecific. Definitive diagnosis is based on ultrasound‐guided pyelocentesis for cytological analysis and bacterial culture, which is an invasive procedure and requires an experienced operator. Moreover, sufficient pelvic dilation is required for an ultrasound‐guided pyelocentesis to be performed, which is not consistently present in all cases of bacterial pyelonephritis.^3^ In the field of human medicine, the presence of both fever and a clinical diagnosis of a urinary tract infection plays a crucial role in indicating bacterial pyelonephritis since individuals with cystitis seldom exhibit fever.^4^ In humans, elevated peripheral white blood cell counts or elevated C‐reactive protein (CRP) are also suggestive of pyelonephritis.^5^, ^6^ Accurate diagnosis of bacterial pyelonephritis remains problematic in veterinary medicine but is crucial considering the therapeutic and prognostic implications.

Positive acute phase proteins are a class of analytes whose blood concentration increases in response to inflammation. Serum amyloid A (SAA) has received increasing attention in the veterinary literature in recent years: increased concentration is associated with nonspecific inflammatory, infectious, or neoplastic processes.^5^, ^6^, ^7^, ^8^ In humans, the utility of acute phase proteins (mostly CRP and procalcitonin) has been evaluated in the diagnostic approach of bacterial pyelonephritis.^9^, ^10^ A recent review concluded that low CRP values appear somewhat useful in ruling out bacterial pyelonephritis in children, but that increased CRP concentration lacked specificity in the diagnosis of upper urinary tract infections.^11^

The aims of our study were (a) to report and compare the clinical, biological, and diagnostic imaging findings in a cohort of cats with suspected or confirmed bacterial pyelonephritis and (b) to evaluate the accuracy of SAA concentration as a marker of bacterial pyelonephritis in cats. We hypothesized that cats with suspected or confirmed bacterial pyelonephritis would have a higher SAA concentration than cats without pyelonephritis but with other diseases of the urinary tract. We also hypothesized that cats with suspected or confirmed bacterial pyelonephritis would have more frequent biochemical or ultrasonographic changes indicative of a renal inflammatory process.

## 2 MATERIALS AND METHODS

Adult cats (>6 months) presented at the veterinary teaching hospital of the École Nationale Vétérinaire d'Alfort between December 2018 and December 2022 were prospectively recruited. All owners gave informed consent to their cat's participation in the study. The study had ethical approval from an established committee (ComERC, number 2020‐05‐24), and animals were cared for according to the principles outlined in the NIH Guide for the Care and Use of Laboratory Animals.

### 2.1 Inclusion criteria

Cats with a history of, or with clinical or biological findings suggestive of upper or lower urinary tract disease including periuria, hematuria, pollakiuria, dysuria, polyuria/polydipsia, azotemia, abnormal renal, or urinary bladder palpation were included. Cats could be included several times if they were presented on several episodes. For being considered as separate episodes, the time lapse between 2 consecutive presentations had to be superior to 3 months, with resolution of the chief complaint and associated clinical abnormalities between the 2 episodes. In all cats, historical and clinical data were recorded, as well as previous treatment administration in the 15 days before presentation (including antibiotic, immunomodulatory, and anti‐inflammatory drugs).

### 2.2 Diagnostic work up

Complete abdominal ultrasonographic examination was performed using a Philips ultrasound machine (Affiniti 50G, Philips medical systems, Amsterdam, the Netherlands) and linear [18‐5 MHz] or microconvex [8‐5 MHz] transducers (Philips medical systems, Amsterdam, the Netherlands). Measurement of renal pelvises was performed by a single operator on still images of the kidneys in transverse plane, using an imaging viewer (Horos 3.0 software, Horos Project, Annapolis, Maryland), and a dedicated imaging workstation (iMac Pro 27' 5K, Apple, Cupertino, California) using a previously described methodology.^12^

Urine was either collected by cystocentesis, sampling from the access port of a subcutaneous ureteral bypass device (SUB), or ultrasound‐guided pyelocentesis using a previously described technique.^13^ Urinalysis included the determination of urine specific gravity using a manually calibrated standard refractometer, semiquantitative analysis through a urinary dipstick, and sediment assessment. For the bacterial culture, a portion of the urine specimen was placed in a sterile vial and promptly transported to the microbiology laboratory or briefly refrigerated at 4°C before transfer. Inoculation of 10 to 100 μL of well‐mixed urine onto blood agar plates was performed, followed by aerobic incubation at 37°C for a maximum of 72 hours. Evaluation of bacterial growth degree and purity was conducted at 24‐hour intervals, with any bacterial growth ≥1000 colonies forming unit (CFU)/mL deemed significant. Matrix‐assisted laser desorption/ionization time‐of‐flight mass spectrometry was used for the identification of bacterial isolates. Antimicrobial susceptibility testing for all isolates was performed using the Kirby‐Bauer disc diffusion method across a range of antimicrobials.

Serum urea and creatinine concentrations were measured in all cats. The assessment of additional hematological or biochemical variables was left to the discretion of the attending clinician.

### 2.3 Group allocation

Cats were allocated into Group 1 and Group 2. Group 1 included cats with confirmed (1a) or highly presumed (1b) bacterial pyelonephritis. Group 2 included cats in which bacterial pyelonephritis was excluded (2a) or judged very unlikely (2b). Confirmation (1a) or exclusion (2a) of bacterial pyelonephritis was based on a positive or negative bacterial culture on urine sampled via ultrasound‐guided pyelocentesis. Presumptive diagnosis of bacterial pyelonephritis (1b) was based on compatible history and clinical evolution (assessed by a consensus between 3 board‐certified internal medicine specialists (GB, CM, MK)) as well as at least 3 of the following criteria: azotemia (defined as serum creatinine concentration above the upper limit of the reference interval), positive bacterial culture on urine collected by cystocentesis or from a SUB access port, hyperthermia (rectal temperature ≥39.5°C), presence of renal pelvic dilation (≥1 mm on transverse ultrasonographic view). Bacterial pyelonephritis was judged unlikely (2b) on the bases of the history and clinical evolution and if <3 of the above criteria were met. Cats on antimicrobial treatment and negative cultures on pyelocentesis were also included in Group 2b providing they fulfilled the aforementioned criteria.

### 2.4 Exclusion criteria

Cats with overt inflammatory, infectious or neoplastic disease detected during the diagnostic work‐up were excluded in order to avoid confounding effect of such diseases on the measurement of SAA. Cats with urethral obstruction were also excluded from the study because previous of literature data reporting increases in acute‐phase proteins in cats with obstructive lower urinary tract disease.^14^ Cats in which SAA quantification was performed more than 48 hours after hospitalization were excluded to avoid the confounding effect of hospital procedures or exposure to nosocomial pathogens on the measurement of SAA. Cats with incomplete medical files were also excluded. Administration of antibiotics, immunomodulatory, or anti‐inflammatory drugs before presentation was not an exclusion criterion.

### 2.5 Measurement of SAA concentration

Measurement of SAA was performed within 48 hours, without freezing the samples. It was performed using a latex agglutination turbidimetric immunoassay that was previously validated in cats (Vet‐SAA Eiken, Eiken Chemical).^15^ Lower limit of detection for this assay was 1 mg/L.

### 2.6 Statistical analyses

All statistical analyses were performed using commercially available software (SAS University Edition). Continuous variables are presented as medians [1st quartile; 3rd quartile]. Hemoglobin concentration, white blood cell, neutrophil, lymphocyte, monocyte and platelet counts, neutrophil to lymphocyte ratio, serum albumin and total proteins concentrations, as well as albumin to protein ratio were compared between groups on a hypothesis‐driven basis by using Kruskal‐Wallis to detect statistically significant differences, followed by pairwise Mann‐Whitney *U* test when relevant. Proportions were compared between groups by using the Chi‐square test (or Fisher's exact test when appropriate). The association between the presence of a SUB and an increase in SAA concentration was evaluated using a liner mixed model accounting for the inclusion of some cats on several occasions. Area under the curve (AUC) of SAA concentration with respect to its ability to detect bacterial pyelonephritis was calculated by receiver operating characteristics (ROC) analysis, performed on the easyROC website (http://biosoft.erciyes.edu.tr/app/easyROC/). Optimal cut‐offs were determined using the generalized Youden index method, which was run using the OptimalCutpoints package from R. Associated sensitivity and specificity are presented with their 95% confidence interval. Positive and negative predictive values were calculated using the observed sensitivity and specificity, and according to 5 hypothetical pretest probabilities. Statistical significance was set at *P* < .05. A power analysis was performed to know how many cats were required to detect a significant difference (at 5% level) in SAA, between cats with and without pyelonephritis, with 95% of statistical power. Based on the hypothesis that SAA (above 12 mg/L) would be increased in 80% of cats with pyelonephritis and in 20% of cats with urinary tract disease other than pyelonephritis: 24 cats (12 cats in each group) were required.

## 3 RESULTS

**FIGURE 1:**
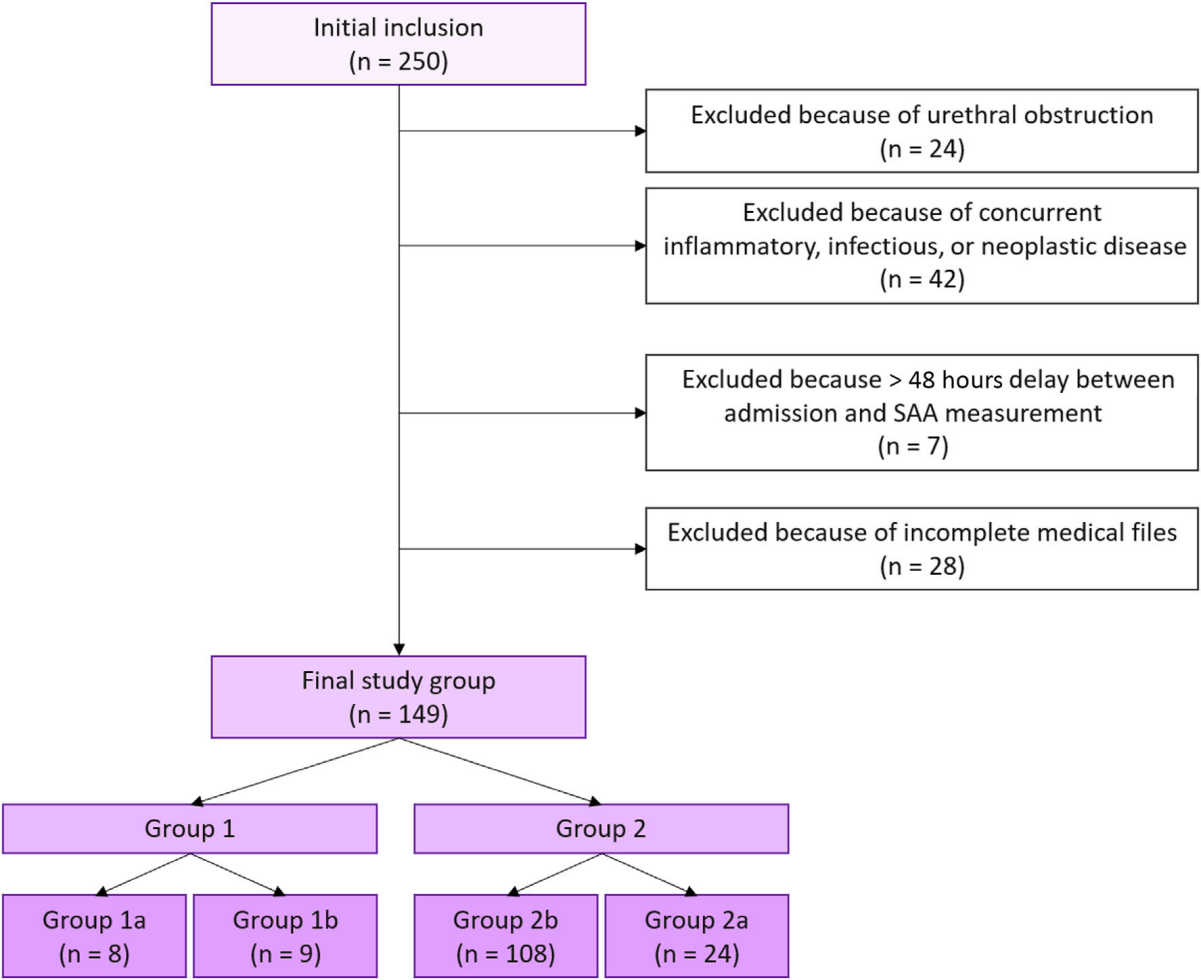
Flow chart representation of the process of inclusion, exclusion, and group allocation in the study sample.

Inclusion criteria were met for 250 observations. Flowchart for exclusion process and group allocation is further described in Figure 1. At the end of the exclusion process, 149 observations were included. A one hundred six cats were included on 1 single observation, 14 cats were included on 2 distinct observations, and 5 cats were included on 3 distinct observations. Inclusions corresponding to cats that were included on multiple occasions all belonged to Group 1b (n = 3) and Group 2b (n = 23). Corresponding chief complaints and clinical signs are reported in Table 1.

**TABLE 1:** Clinical signs and chief complaints of cats between groups, in the study sample.

		Group 1a (n = 8)		Group 1b (n = 9)		Group 2a (n = 19)		Group 2b (n = 113)	
		n	%	n	%	n	%	n	%
Chief complaints	Decreased appetite	6	75	5	56	14	58	33	29
	Lethargy	3	38	2	22	6	25	16	14
	Weight loss	0	0	0	0	1	4	9	8
	Vomiting	3	38	2	22	9	38	12	11
	Constipation	0	0	0	0	0	0	1	1
	Diarrhea	0	0	0	0	0	0	0	0
	PUPD	0	0	0	0	1	4	6	5
	Stranguria	0	0	2	22	0	0	3	3
	Pollakiuria	0	0	2	22	0	0	3	3
	Malodorous urine	0	0	0	0	0	0	1	1
	Hematuria	1	13	0	0	0	0	6	5
	Periuria	0	0	0	0	0	0	1	1
	Painful attitude	0	0	0	0	0	0	1	1
	Alopecia, pruritus, squamosis	0	0	0	0	1	4	2	2
Routine check up	Routine SUB follow‐up	0	0	1	11	1	4	29	26
	Routine CKD follow up	0	0	0	0	4	17	11	10

*Note*: Group 1a: cats with confirmed pyelonephritis, Group 1b: cats with pyelonephritis being judged likely, Group 2a: cats with pyelonephritis being excluded, Group 2b: cats with pyelonephritis being judged unlikely.

Abbreviations: CKD, chronic kidney disease; PUPD, polyuria and polydipsia; SUB, subcutaneous ureteral bypass.

Median duration of clinical signs before presentation was significantly shorter in cats from Group 1 (48 days [24; 72]) than Group 2 (168 days [84; 1116], *P* < .001). Nine cats in the study sample had received antibiotics before the inclusion and they all belonged to Group 2b, in which they represented 8% of the total effective within this group. No cat had received immunomodulatory or anti‐inflammatory drug before presentation. Methods for urine collection and results of urinary bacterial culture are reported in Table 2. Clinical, biochemical and urinalysis findings as well as ultrasonographic findings among groups are described in Tables 3a and 3b. Final presumptive diagnoses for cats in Groups 2a and 2b are reported in Table 4.

**TABLE 2:** Method for urine collection in the different groups of the study sample, and results of urinary bacterial culture.

		Group 1a (n = 8)		Group 1b (n = 9)		Group 2a (n = 19)		Group 2b (n = 113)	
		n	%	n	%	n	%	n	%
	Pyelocentesis	8	100			19	100	5	4
	Cystocentesis			7	78			108	96
	SUB access port puncture			2	22				
Species (number of isolates/number of animals with bacteriuria in the group, %)	*Escherichia coli*	7/8	12	5/10	50			1/12	8
	*Enterococcus faecalis*			2/10	20				
	*Peudomonas aeruginosa*	1/8	2	1/10	10			5/12	42
	*Staphylococcus epidermidis*			1/10	10			2/12	17
	*Klebsiella pneumoniae*							2/12	17
	*Enterococcus faecium*			1/10	10				
	*Staphylococcus felis*							1/12	8
	*Proteus mirabilis*							1/12	8
	Total of isolates	8		10		0		12	

*Note*: Group 1a: cats with confirmed pyelonephritis, Group 1b: cats with pyelonephritis being judged likely, Group 2a: cats with pyelonephritis being excluded, Group 2b: cats with pyelonephritis being judged unlikely.

Abbreviation: SUB, subcutaneous ureteral bypass.

**TABLE 3a:** Hematological, biochemical, and medical imaging data in the different groups of the study sample, represented as medians [1st quartile; 3rd quartile].

	Group 1a (n = 8)	Group 1b (n = 9)	Group 2a (n = 19)	Group 2b (n = 113)	Reference interval	*P* value	N =
Sex (n, %)							149
Intact female	0 (0%)	0 (0%)	0 (0%)	4 (4%)			
Intact male	0 (0%)	0 (0%)	0 (0%)	2 (2%)			
Neutered female	7 (88%)	8 (89%)	9 (47%)	59 (52%)			
Neutered male	1 (13%)	1 (11%)	10 (53%)	48 (42%)			
Age (years)	4.5 [3; 8.5]	11 [10; 12]	8 [6; 10]	8 [5; 12]			149
Rectal temperature (°C)	39.2 [38.7; 40.1]	39.3 [38.1; 39.8]	38.4 [38.2; 38.9]	38.2 [38; 38.5]			149
Urea (mmol/L)	31.0 [19.0; 50.9]	25.9 [18.4; 38.7]	28.2 [14.9; 38.2]	16.1 [11.5; 33.7]	6.7‐13.4		149
Creatinine (μmol/L)	354 [216; 590]	350 [334; 533]	424 [221; 1106]	248 [159; 425]	46‐157		149
Maximal renal pelvis diameter (mm)	12.5 [7.5; 17.5]	2.7 [2; 4]	6.5 [4.1; 10.0]	2.0 [0; 4]			149
Maximal ureteral diameter (mm)	4.5 [4.1; 7.5]	2.1 [1.8; 2.7]	3.4 [2.0; 6.0]	1.3 [0; 2.7]			149
Hemoglobin (g/dL)	10.4 [7; 16.3]	9 [6.3; 12.4]	9.6 [7.2; 11.4]	8.9 [7.9; 11.0]	9.8‐16.9	*P* = .97	68
White blood cells (/mm^3^)	17 480 [11 575; 26 605]	9630 [6820; 15 210]	10 550 [8735; 11 420]	10 905 [7050; 14 520]	3700‐18 660	*P* = .38	68
Neutrophils (/mm^3^)	14 395 [9445; 22 454]	7008 [5440; 10 496]	7540 [6230; 8680]	6420 [4310; 11 200]	1450‐9620	*P* = .19	68

**TABLE 3b:** Hematological, biochemical, and medical imaging data in the different groups of the study sample, represented as percentage of the study sample with abnormal results.

		Group 1a (n = 8)	Group 1b (n = 9)	Group 2a (n = 19)	Group 2b (n = 113)	*P* value
Hyperthermia (n, %)	Number with abnormalities/number included (%)	3 (38%)	4 (44%)	1 (5%)	1 (1%)	
Increased serum creatinine concentration (n, %)		7 (88%)	9 (100%)	18 (95%)	93 (82%)	
Renal pelvis dilation (n, %)		8 (100%)	9 (100%)	19 (100%)	80 (71%)	
Bacteriuria (n, %)		8 (100%)	8 (89%)	0 (0%)	12 (11%)	
Ureteral dilation (n, %)		8 (100%)	9 (100%)	18 (95%)	73 (64%)	
Upper urinary tract lithiasis (n, %)		7 (88%)	7 (78%)	15 (79%)	81 (72%)	
Lower urinary tract lithiasis (n, %)		2 (25%)	2 (22%)	7 (37%)	34 (31%)	
Perirenal hyperechogenicity (n, %)		5 (63%)	4 (44%)	6 (32%)	42 (37%)	*P* = .47
Pelvic or renal hyperechogenicity (n, %)		2 (25%)	5 (56%)	6 (32%)	32 (28%)	*P* = .38
Urine dipstick:blood	Number with abnormalities/number evaluated (%)	5/8 (63%)	6/9 (66%)	13/18 (72%)	79/99 (79%)	
Urine dipstick:proteins		7/8 (88%)	7/9 (78%)	14/18 (78%)	60/99 (61%)	
Hyperglobulinemia		3/3 (100%)	4/4 (100%)	10/19 (67%)	69/91 (76%)	*P* = .57
Leukocytosis		2/4 (50%)	0/6 (0%)	0/8 (0%)	5/50 (10%)	*P* = .10
Neutrophilia		3/4 (75%)	2/6 (33%)	1/8 (13%)	17/49 (35%)	*P* = .22
Monocytosis		2/4 (50%)	1/6 (17%)	0/8 (0%)	9/49 (18%)	*P* = .18

*Note*: Supplementary data is available in a corresponding file. Group 1a: cats with confirmed pyelonephritis, Group 1b: cats with pyelonephritis being judged likely, Group 2a: cats with pyelonephritis being excluded, Group 2b: cats with pyelonephritis being judged unlikely.

Abbreviations: ALP, alkaline phosphatase; ALT, alanine aminotransferase; IF, intact female; IM, intact male; NF, neutered female; NM, neutered male.

**TABLE 4:** final diagnoses in Group 2b and 2a (ie, in cats with no pyelonephritis) and corresponding SAA concentrations (results are presented as medians [1st quartile; 3rd quartile] or as individual values when n = 1).

		Group 2a			Group 2b		
		n	%	SAA concentration (mg/L); range: [0.8‐10.6]	n	%	SAA concentration (mg/L); range: [0.4‐270.9]
Lower urinary tract disease	Bacterial cystitis				4	4	5.6 [2.9; 24.2]
	Lithiasis‐associated cystitis				2	2	47.9 [2.1; 93.7]
	Subclinical bacteriuria				1	1	2.1
Ureteral disease	Ureteral obstruction	14	74	4.5 [1.8; 5.6]	14	12	11.4 [4.5; 77.1]
	Ureteral rupture with uroretroperitoneum	1	5	0.9		0	
Renal disease	Stable CKD without bacteriuria	3	16	5.2 [3.0; 8.4]	64	57	4.7 [3.0; 7.0]
	Stable CKD + subclinical bacteriuria		0		6	5	7.7 [3.2; 9.2]
	AKI of unknown origin	1	5	2.4	20	18	8.3 [4.2; 23.6]
	Others		0		2	2	16.8 [10.8; 22.8]
Total		19	100		113	100	

*Note*: Group 2a: cats with pyelonephritis being excluded, Group 2b: cats with pyelonephritis being judged unlikely.

Abbreviations: AKI, acute kidney injury; CKD, chronic kidney disease.

Group 1a and 1b (ie, cats with confirmed or presumed bacterial pyelonephritis, n = 17) were all domestic shorthair cats. Only 1/17 (6%) cats did not present with azotemia at admission. Prevalence of hyperthermia (rectal temperature >39.5°C) was 41% in Group 1 (7/17 cats) and 2% in Group 2 (3/132 cats). Complete blood count results were available in 46% of cats (68/149 cats). Exhaustive biochemistry panel has been evaluated in 30% of cats (45/149). Prevalence of hyperglobulinemia, leukocytosis, neutrophilia and monocytosis were not significantly different between Group 1 and 2 (*P* = .57; .10; .22; and .18 respectively). Neutrophil lymphocyte ratio was not significantly different between the 2 groups (*P* = .26).

All cats with confirmed bacterial pyelonephritis (Group 1a) presented single bacterial isolates whereas multiple bacterial isolate infections were detected in 1/10 (10%) episodes within Group 1b. Each bacterial culture in Group 2b resulted in the isolation of a single bacterial isolate, and all these episodes were considered to be subclinical bacteriuria or bacterial cystitis.

**FIGURE 2:**
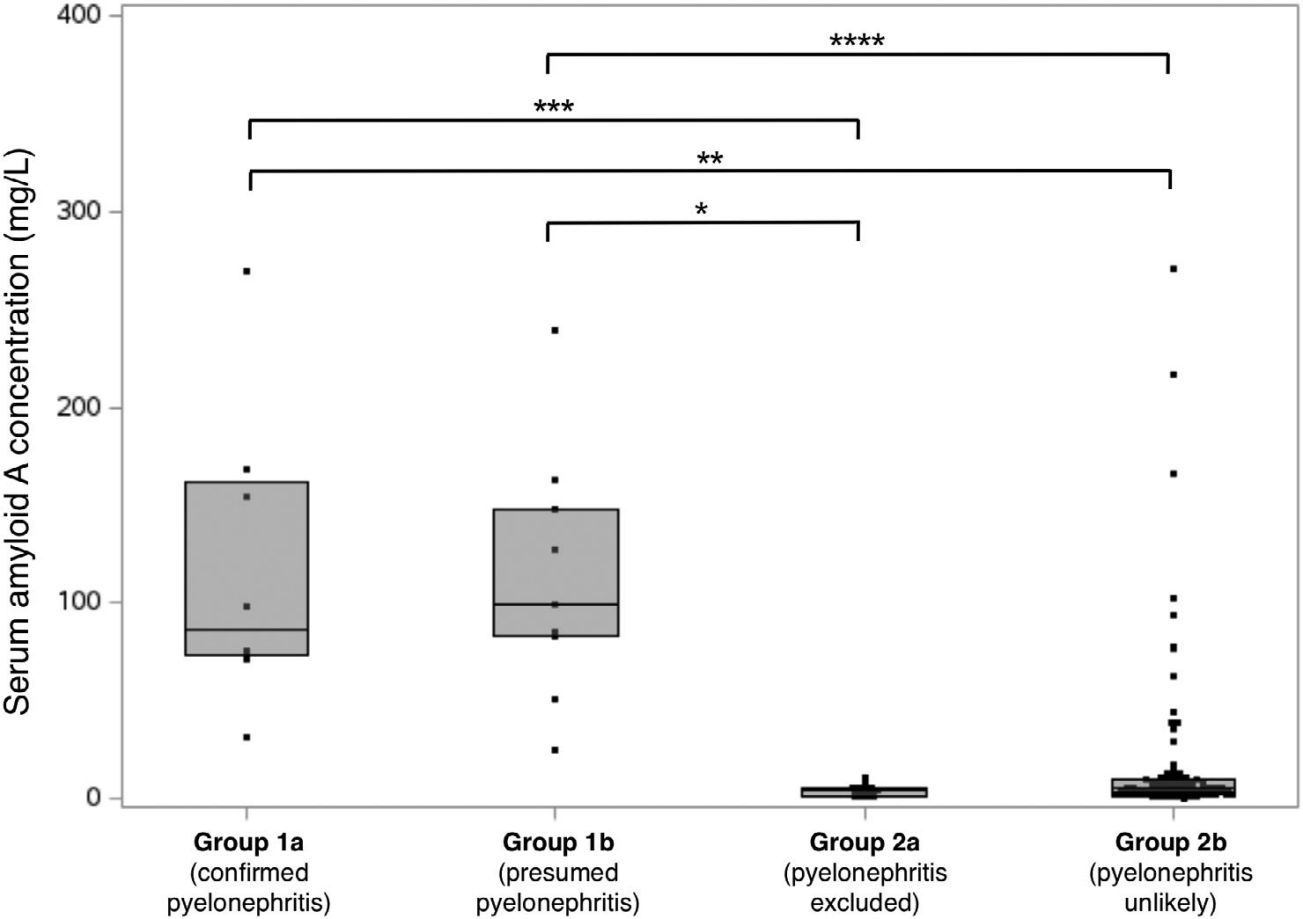
Box plots representing the concentration of Serum Amyloid A (SAA) in the different groups of the study sample. Group 1a and Group 2a: cats with confirmed/excluded pyelonephritis based on positive/negative bacterial culture on urine collected by pyelocentesis, respectively. Group 1b and 2b: cats with pyelonephritis being judged likely or unlikely based on clinical and biological criteria (see material and methods).

Distribution of SAA concentrations in the different groups is reported in Figure 2. SAA concentrations were above the limit of detection in all samples. Median SAA concentration was significantly higher in Group 1a (86.8 mg/L [73.3; 161.5]) than in Group 2a (4 mg/L [1.8; 5.6], *P* < .001) and in Group 2b (5.4 mg/L [3.1; 9.7], *P* < .001). It was also significantly higher in Group 1b (98.8 mg/L [83.1; 147.3]) than in Group 2b (*P* < .001) and Group 2a (*P* < .001). Other differences were not significant. There was no statistical association between the presence of a SUB and an increase in SAA concentration (*P* = .45).

Serum Amyloid A concentration was increased above the upper limit of the reference interval in 8/8 (100%) cats in Group 1a, 9/9 (100%) cats in Group 1b, 0/19 (0%) cats in Group 2a and 20/113 (17%) cats in Group 2b. Proportion of cats with increased SAA concentration was significantly higher in Group 1a than in Group 2a (*P* < .001) and 2b (*P* < .001). It was also significantly higher in Group 1b than in Group 2a (*P* < .001) and 2b (*P* < .001). Other differences were not statistically significant.

**FIGURE 3:**
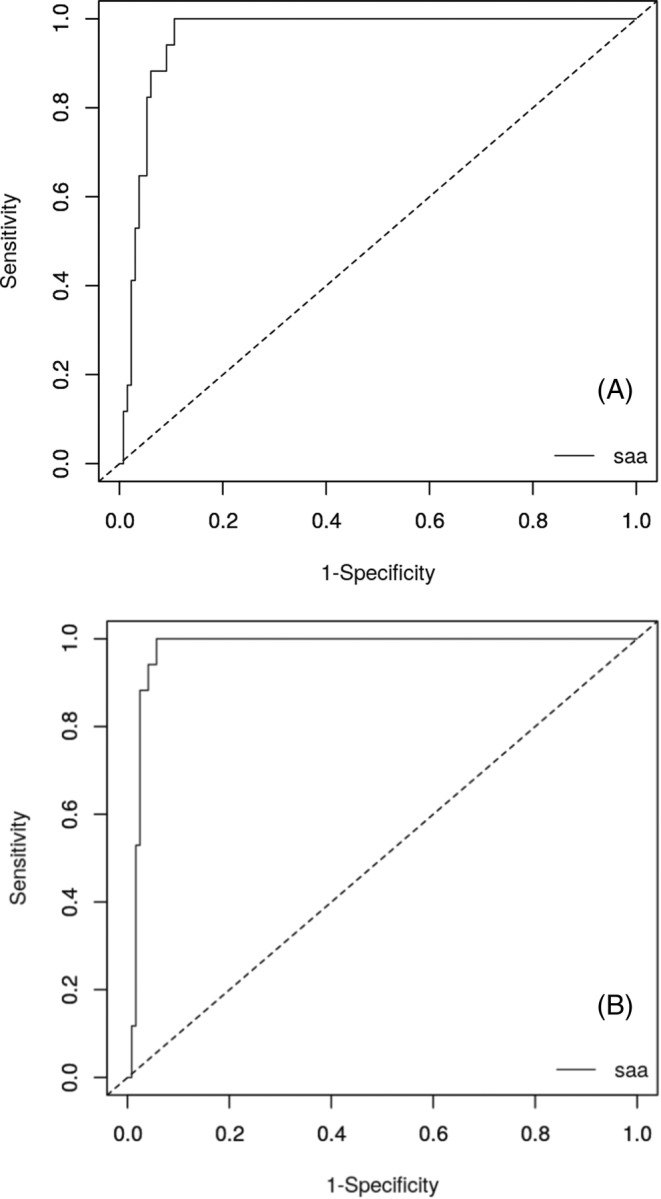
(A) Receiving Operating Characteristics (ROC) curve for the ability of SAA concentration to detect pyelonephritis, that is, to differentiate cats from Group 1 (cats with confirmed or presumed pyelonephritis) and 2 (cats with pyelonephritis being excluded or judged unlikely). (B) same curve after exclusion of cats that had received antibiotics before presentation.

When Group 1 and 2 were considered as single groups, the AUC for the ability of SAA concentration to detect bacterial pyelonephritis was 0.96 (Figure 3A). Optimal diagnostic cut‐off for SAA concentration, determined by the generalized Youden index method, was 51.3 mg/L. This cut off yielded a sensitivity of 88% (95% confidence interval: [64%; 99%]) and a specificity of 94% (95% confidence interval: [88%; 97%]). Positive and negative predictive values, calculated using the observed sensitivity and specificity and 5 hypothetical pretest probabilities, are represented in Table 5. When cats that had received antibiotics before presentation were excluded from the ROC analysis, AUC increased to 0.98 (Figure 3B). The optimal diagnostic cut‐off was 25 mg/L, yielding a sensitivity of 100% (95% confidence interval: [94%; 100%]) and a specificity of 94% (95% confidence interval: [92%; 98%]).

**TABLE 5:** Positive and negative predictive values calculated from the observed sensitivity and specificity, and based on 5 hypothetical pretest probabilities (5%‐50%).

5%		10%		20%		30%		50%	
PPV	NPV	PPV	NPV	PPV	NPV	PPV	NPV	PPV	NPV
44%	99%	62%	99%	79%	97%	86%	95%	94%	89%

Abbreviations: NPV, negative predictive value; PPV, positive predictive value.

## 4 DISCUSSION

Our study investigated the usefulness of a readily available biological marker in the diagnosis of bacterial pyelonephritis in cats. The diagnosis of upper urinary tract infections in cats continue to be a common and crucial concern in clinical practice, but it remains rather challenging. There is a scarcity of literature data on this condition. The most recent case series includes 17 histologically confirmed cases of feline bacterial pyelonephritis, and has only been reported as an abstract.^16^ It is likely that upper urinary tract infections in cats are underdiagnosed in some contexts, and overdiagnosed in some others. Pyelonephritis was reported in 8% of cats presenting an acute‐on‐chronic kidney disease in a recent study.^1^ Diagnosing pyelonephritis in the absence of azotemia in 1 cat in the current study demonstrates the challenge of diagnosis of pyelonephritis in cats.

In feline practice, measurement of SAA is increasingly used as a marker of active inflammatory or neoplastic processes and procalcitonin is unfortunately not easily available.^17^, ^18^, ^19^, ^20^, ^21^, ^22^, ^23^, ^24^ Although SAA has been used as a marker of bacterial pyelonephritis in the veterinary literature,^25^ its diagnostic accuracy had not yet been investigated. In our study, we have identified that SAA is a valuable tool in the diagnosis of feline bacterial pyelonephritis, as it is more commonly increased in cats with upper urinary tract infections than in cats with other urinary tract or renal diseases. We have found SAA to be more sensitive than specific in the diagnosis of feline bacterial pyelonephritis. In other words, bacterial pyelonephritis is highly unlikely in an adult cat if the SAA concentration is low, but a high SAA concentration can be caused by a bacterial pyelonephritis or another disease responsible for an inflammatory response. In Group 2a, 5 cats had an increased SAA concentration (4 of them having a SAA concentration >25 mg/L) with negative bacterial culture on urine collected via pyelocentesis and no overt inflammatory disease. However, these cats were receiving ongoing antibiotic treatment at the time of sampling and it is thus possible that they were misclassified. Interestingly, when cats that had received antibiotic treatment before presentation were excluded from the statistical analysis, the sensitivity of SAA in the diagnosis of pyelonephritis increased to 100%, which means that a SAA within in the reference interval ruled out bacterial pyelonephritis with excellent confidence levels in this subset of our study sample. Whether SAA measurement could be used as a better marker for bacterial pyelonephritis than urinary bacterial culture in cats that have received antibiotics before presentation remains to be confirmed. It is also important to note that no hematological or biochemical variable other than SAA were significantly different between groups. Neutrophilia (detected in 75% of cats in Group 1a, and between 30% and 36% in other groups) was the most common hematologic abnormality in cats with pyelonephritis, followed by monocytosis that was observed in 50% of cats in Group 1a while it was less frequent in other groups (9%‐17%). We think that this may be attributable to a lack of statistical power, as previous studies have identified monocytosis to be the sole hematological modification that was associated with bacterial pyelonephritis in cats with ureteral obstruction.^26^

A reliable diagnosis of bacterial pyelonephritis is crucial for therapeutic considerations. Fluoroquinolones are frequently recommended for the management of upper urinary tract infections, but in some European countries, their use is highly restricted because of antibiotic resistance concerns and the prescriber must provide tangible diagnostic evidence.^27^, ^28^ Use of these antibiotics should be limited to cases where alternatives are less effective. Treatment duration for pyelonephritis may be longer than for cystitis, but recent recommendations suggest 7 to 14 days may be sufficient for acute cases, revising the previous 4 to 6 weeks guideline.^29^

Overall, diagnostic criteria for bacterial pyelonephritis in cats remain poorly defined. The International Society for Companion Animals Infectious Diseases clearly states that the diagnosis of upper urinary tract infections is a challenge. It should be based on positive bacterial urine culture accompanied by systemic signs (fever, lethargy, polyuria/polydipsia), renal pain, azotemia, cylindruria, and peripheral neutrophilia.^27^ However, this approach has several limitations. First, these criteria are mostly present in cases of acute pyelonephritis and not necessarily in chronic forms, in which systemic signs, fever and renal pain may be absent, as it is reported in humans.^30^ Second, the classic features of pyelonephritis are not present in all animals: for instance, the proportion of cats with bacterial pyelonephritis presenting with hyperthermia or renal pain is not clearly known, but may be as low as 30%. Indeed, in 17 cases of histologically confirmed bacterial pyelonephritis, only 3 cats presented with renal pain and 2 cats presented with pyrexia.^16^ In our cohort, only 38% of cats with confirmed upper urinary tract infections had hyperthermia. However, it is essential to acknowledge the potential bias in these figures, as our data originates from a referral cohort, which could influence the disease presentation. Thirdly, detection of azotemia may not be a reliable feature of upper urinary tract infections in cats, as bacterial pyelonephritis frequently occurs as a consequence to or in association with an underlying renal disease and/or ureteral obstruction. In experimental models from the 1950s, pyelonephritis could not be produced without inducing some form of renal injury, such as obstruction, massage, or trauma to the kidney, which supports the latter claim.^27^ Fourth, subclinical bacteriuria may occur in 6% to 30% of cats over 6 years old, and up to 30% of cats with chronic kidney disease have positive urinary bacterial culture.^31^, ^32^, ^33^ Lastly, discrepancies between results of bacterial culture of urine collected by cystocentesis or pyelocentesis may occur, especially in the context of ureteral obstruction.^34^ Caution should thus be exercised in interpreting negative bacterial culture results from urine collected via cystocentesis in cats with ureteral obstruction or ongoing antimicrobial treatment. Similarly, positive results warrant careful consideration, as the isolated bacterial species may differ from those present in the renal pelvises. Unfortunately, no cat in our cohort underwent concurrent pyelocentesis and cystocentesis to further document this phenomenon.

Strong evidence‐based data about the usefulness of urinary tract ultrasonography for the diagnosis of pyelonephritis is lacking. Although hyperechogenicity of the renal parenchyma, pelvic dilation, and abnormal retroperitoneal echogenicity are generally considered to be suggestive of bacterial pyelonephritis, no study has attempted to clearly identify the ultrasonographic features of feline pyelonephritis—probably because such a study would be difficult to design because of the lack of diagnostic consensus.^3^, ^12^ For these reasons, our group allocation algorithm was constructed with exclusive regard to the presence or absence of renal pelvis dilation. However, it is likely that bacterial pyelonephritis sometimes occurs without pyelectasia,^3^ and perhaps even without renal ultrasonographic abnormalities. It would have been interesting to evaluate other ultrasonographic criteria such as symmetry of the renal pelvis and distortion of the pelvis recesses. Further studies evaluating the diagnostic performance of ultrasonography for pyelonephritis in cats are needed. We suggest that future studies addressing feline upper urinary tract infections consider incorporating SAA measurement as an additional criterion to enhance the precision of individual classification.

Our study contributes additional data to the growing evidence supporting a high prevalence of urolithiasis in cats with bacterial pyelonephritis. Interestingly, concurrent ultrasonographic detection of upper or lower uroliths was detected in more than half of the cases of presumed or confirmed bacterial pyelonephritis in our cohort. We therefore mainly suspect calcium oxalate stones. Upper urinary lithiasis has been recognized as a risk factor for the development of bacterial pyelonephritis in humans by causing urine flow obstruction, mechanical irritation of the urothelium and by providing support for bacteria attachment.^35^ Although none of these uroliths have been submitted to spectrophotometric analysis, struvite stones seem unlikely given the low prevalence of urease‐producing bacteria.

A large proportion of cats in our study had a SUB at the time of inclusion. We acknowledge that the presence of a SUB may confound the diagnostic approach of bacterial pyelonephritis, because SUB devices can be associated with mild pelvic dilations even in the absence of active renal obstructive or inflammatory disease.^36^ Moreover, even though no association between the presence of a SUB and an increase in SAA concentration was identified in our study sample, the effect of long‐term SUB devices on SAA concentration is currently unknown. It is thus possible that inclusion of such cats may have induced classification bias. Our choice to include cats with SUB was motivated by the will to illustrate real clinical practice as closely as possible. Indeed, 20% to 36% of cats with SUB eventually develop bacteriuria during the postoperative follow‐up period.^34^, ^37^, ^38^ Whether this bacterial infection stays confined to the urinary bladder and SUB device or can affect the renal parenchyma and participate in the progression of kidney damage requires further investigation. It is currently difficult, not to say impossible, to differentiate bacterial pyelonephritis from bacterial cystitis in cats with a SUB in place. We believe the results of our study provide a tool to differentiate these entities.

Our study had several limitations. Few cats were included in Group 1a and 1b compared with Group 2a and 2b. The repeated inclusion of some animals raises some concerns. Nonetheless, a sensitivity analysis was conducted to examine the potential impact of this inclusion process on the diagnostic accuracy of SAA. Interestingly, the results were not clinically different when compared with those obtained with the same analysis performed when cats were included on just their first visit. This observation is likely attributable to the fact that cats that were included on multiple episodes mostly originated from Group 2b, which comprises the largest number of individuals. Inclusion of cats that had received medications within 15 days before enrolment can be questioned, however, although none of the cats had received anti‐inflammatory or immunomodulatory drugs before presentation, a significant proportion had been prescribed antibiotics. All cats that had received antibiotics belonged to Group 2b, and they represented 8% of the total effective within this group. Although this method offers the benefit of mirroring the typical experiences of cat practitioners, it could potentially introduce bias: the prescription of antibiotics before enrollment may lead to false negative results when exploring pyelonephritis, by potentially masking the presence of bacteriuria or fever, thus leading to inaccurate diagnostic outcomes. This masking effect could obscure the true prevalence or severity of pyelonephritis in the study sample, introducing bias into the results. Consequently, we conducted an additional statistical analysis: when focusing solely on cats that had not received antibiotics before presentation, diagnostic accuracy of SAA to diagnose pyelonephritis increased slightly. Cats were also excluded if they showed clear signs of concurrent inflammatory, infectious, or neoplastic disease. By making the case selection too restrictive, this could potentially introduce bias, resulting in an overestimation of test accuracy. Furthermore, our group allocation algorithm to classify cats into Groups 1b and 2b was not perfect, because it relies on an estimated likelihood of bacterial pyelonephritis rather than on a definitive diagnosis.

The confirmation of bacterial pyelonephritis in Group 1a relied on obtaining urine by pyelocentesis, which may have induced a selection bias as cats with bacterial pyelonephritis without a sufficiently large pelvic dilation to allow pyelocentesis or facing a less confident radiologist to perform pyelocentesis in a small pelvis would have been misclassified. Inclusion in Group 1b partly relied on the identification of renal pelvis dilation. Our cohort of cats in Group 1 may therefore not be representative of all cats with bacterial pyelonephritis with regards to the severity of the pelvic dilation. If the severity of renal pelvis dilation was associated with disease severity, it is possible that cats with bacterial pyelonephritis and minimal renal pelvic dilations had lower SAA concentrations than cats with larger pelvic dilations. This remains to be investigated. Diagnosing pyelonephritis using pyelocentesis might also overestimate the prevalence of concomitant nephrolithiasis, urolithiasis and ureteral obstruction. Diagnosis of pyelonephritis concurrently with ureterolithiasis may also be biased by the referral cohort, given that primary care vets may be more likely to refer where they have identified renal pelvis dilation. Lastly, paraclinical data (results from complete blood counts or blood smear examinations) were lacking in some cats, in such a way that comparison of the diagnostic accuracy of SAA with the presence of a neutrophilic left shift or monocytosis was limited.

## 5 CONCLUSION

Definitive diagnosis of bacterial pyelonephritis remains a challenge in cats, since presenting clinical signs lack specificity, and confirmatory testing relies on ultrasound‐guided pyelocentesis, which is invasive and sometimes impossible when pelvic dilation is absent. Development of an easily accessible, accurate diagnostic tool for the diagnosis of bacterial pyelonephritis and its differentiation from other urinary tract or renal diseases such as ureteral obstructions, bacterial cystitis, or subclinical bacteriuria, is needed. Our study showed that SAA concentration is higher in cats with confirmed or presumed bacterial pyelonephritis than in cats in which bacterial pyelonephritis was excluded or judged unlikely. Serum Amyloid A measurement using a threshold of (51.3 mg/L) seems sensitive in the diagnosis of pyelonephritis in cats, and could therefore be used as an exclusion test. However, SAA measurement showed several limitations as it is not specific for bacterial pyelonephritis, and can be increased because of other inflammatory diseases.

## CONFLICT OF INTEREST DECLARATION

Authors declare no conflict of interest.

## OFF‐LABEL ANTIMICROBIAL DECLARATION

Authors declare no off‐label use of antimicrobials.

## INSTITUTIONAL ANIMAL CARE AND USE COMMITTEE (IACUC) OR OTHER APPROVAL DECLARATION

École Nationale Vétérinaire d'Alfort ComERC Ethics Committee (internal number: 2020‐05‐24) approval. The work involved the use of serum and urine samples obtained from non‐experimental, owned animals. The study had prior ethical approval from an established committee as stated in the manuscript. Written informed consent was obtained from the owner or legal custodian of all animals described in this work for all procedures undertaken.

## HUMAN ETHICS APPROVAL DECLARATION

Authors declare human ethics approval was not needed for this study.

## Supporting information


**Table S1.** Hematological and biological data among the groups from the study population. Group 1a: cats with confirmed pyelonephritis, Group 1b: cats with pyelonephritis being judged likely, Group 2a: cats with pyelonephritis being excluded, Group 2b: cats with pyelonephritis being judged unlikely.
