# Supplementary material for: Usefulness of serum amyloid A for the diagnosis of pyelonephritis in cats: A prospective evaluation
Source: J Vet Intern Med. 2024 Apr 26;38(3):1542–52. doi: 10.1111/jvim.17082 (PMC11099735; doi:10.1111/jvim.17082)
Supplement: Supplementary file 1 — Table S1. Hematological and biological data among the groups from the study population. Group 1a: cats with confirmed pyelonephritis, Group 1b: cats with pyelonephritis being judged likely, Group 2a: cats with pyelonephritis being excluded, Group 2b: cats with pyelonephritis being judged unlikely. [file JVIM-38-1542-s001.pdf]

Supplementary Table 1: hematological and biological data among the groups from the study population. Group 1a: cats with confirmed pyelonephritis, Group 1b: cats with pyelonephritis being judged likely, Group 2a: cats with pyelonephritis being excluded, Group 2b: cats with pyelonephritis being judged unlikely.

	Group 1a	Group 1b	Group 2a	Group 2b	Reference interval	P value
Lymphocytes (/mm3)	1,325 [1,025; 2,888]	1,570 [1,230; 1,706]	1,655 [1,420; 2,910]	1,390 [1,050; 2,680]	1,180 - 10,360	*P* = .75
Monocytes (/mm3)	1,391 [431; 2,165]	405 [261; 525]	245 [188; 340]	350 [240; 640]	90 - 820	*P* = .11
Eosinophils (/mm3)	220 [105; 289]	248 [100; 300]	370 [180; 500]	240 [120; 145]	160 - 1,810	*P* = .50
Neutrophil to lymphocyte ratio	9.1 [7.3; 10.8]	5.7 [4.4; 9.3]	4.9 [3.1; 5.4]	3.6 [2.0; 8.8]		*P* = .26
Platelets (/mm3)	235,000 [205,500; 261,500]	143,000 [50,000; 163,000]	293,000 [262,000; 324,000]	232,000 [122,000; 319,000]	72,000 - 457,000	*P* = .061
Albumin (g/L)	29 [28; 31]	31 [30; 32]	30 [29; 32]	30 [29; 32]	25 - 35	*P* = .76
Total proteins (g/L)	77 [74; 82]	79 [68; 81]	72 [63; 79]	75 [70; 78]	64 - 96	*P* = .31
Albumin to globulin ratio	0.58 [0.51; 0.67]	0.62 [0.54 - 0.69]	0.76 [0.68; 0.88]	0.69 [0.63; 0.79]	> 0.8	*P* = .062
ALT (IU/L)	17 [10; 24]	60 [51; 120]	56 [34; 86]	40 [25; 56]	15 - 123	
ALP (IU/L)	15 [10; 20]	25 [16; 55]	41 [31; 51]	38 [17; 68]	22 - 187	
Sodium (mmol/L)	151 [147; 160]	150 [145; 151]	155 [151; 156]	154 [151; 157]	150 - 165	
Potassium (mmol/L)	4.2 [3.3; 4.6]	4.6 [3.8; 5.6]	4.5 [4.2; 4.8]	4.4 [4; 4.9]	3.6 - 5.5	
Ionized calcium (mmol/L)	1.21 [1.135; 1.32]	1.26 [1.21; 1.31]	1.26 [1.20; 1.33]	1.27 [1.2; 1.35]	1.1 - 1.4	
